# A pilot randomized controlled trial to promote healthful fish consumption during pregnancy: The Food for Thought Study

**DOI:** 10.1186/1475-2891-12-33

**Published:** 2013-03-15

**Authors:** Emily Oken, Lauren B Guthrie, Arienne Bloomingdale, Deborah N Platek, Sarah Price, Jess Haines, Matthew W Gillman, Sjurdur F Olsen, David C Bellinger, Robert O Wright

**Affiliations:** 1 Department of Population Medicine, Harvard Medical School and the Harvard Pilgrim Health Care Institute, 133 Brookline Avenue, Boston, MA, 02215, USA; 2 Channing Laboratory, Brigham and Women’s Hospital, Boston, USA; 3 Boston Children’s Hospital, Boston, USA; 4 Department of Obstetrics, Harvard Vanguard Medical Associates, Boston, USA; 5 Department of Family Relations and Applied Nutrition, University of Guelph, Guelph, Canada; 6 Centre for Fetal Programming, Statens Serum Institut, Copenhagen, Denmark; 7 Department of Nutrition, Harvard School of Public Health, Boston, USA; 8 Mount Sinai School of Medicine, New York City, USA

**Keywords:** Fish, Pregnancy, Nutrition, Mercury, Omega-3 fatty acid, Docosahexaenoic acid (DHA)

## Abstract

**Background:**

Nutritionists advise pregnant women to eat fish to obtain adequate docosahexaenoic acid (DHA), an essential nutrient important for optimal brain development. However, concern exists that this advice will lead to excess intake of methylmercury, a developmental neurotoxicant.

**Objective:**

Conduct a pilot intervention to increase consumption of high-DHA, low-mercury fish in pregnancy.

**Methods:**

In April-October 2010 we recruited 61 women in the greater Boston, MA area at 12–22 weeks gestation who consumed <=2 fish servings/month, and obtained outcome data from 55. We randomized participants to 3 arms: Advice to consume low-mercury/high-DHA fish (n=18); Advice + grocery store gift cards (GC) to purchase fish (n=17); or Control messages (n=20). At baseline and 12-week follow-up we estimated intake of fish, DHA and mercury using a 1-month fish intake food frequency questionnaire, and measured plasma DHA and blood and hair total mercury.

**Results:**

Baseline characteristics and mean (range) intakes of fish [21 (0–125) g/day] and DHA from fish [91 (0–554) mg/d] were similar in all 3 arms. From baseline to follow-up, intake of fish [Advice: 12 g/day (95% CI: -5, 29), Advice+GC: 22 g/day (5, 39)] and DHA [Advice: 70 mg/d (3, 137), Advice+GC: 161 mg/d (93, 229)] increased in both intervention groups, compared with controls. At follow-up, no control women consumed >= 200mg/d of DHA from fish, compared with 33% in the Advice arm (p=0.005) and 53% in the Advice+GC arm (p=0.0002). We did not detect any differences in mercury intake or in biomarker levels of mercury and DHA between groups.

**Conclusions:**

An educational intervention increased consumption of fish and DHA but not mercury. Future studies are needed to determine intervention effects on pregnancy and childhood health outcomes.

**Trial registration:**

Registered on clinicaltrials.gov as NCT01126762

## Introduction

In recent years, there has been active interest in reconciling the potential benefits and harms of prenatal fish consumption to provide optimal fish consumption guidance [1]. Fish, including finfish and other seafood, are the primary dietary source for elongated n-3 polyunsaturated fatty acids (PUFA) [2]. Adequate intake of n-3 (also known as omega-3) PUFA, particularly docosahexaenoic acid (DHA), is essential for optimal fetal neurodevelopment, and may also protect against other adverse perinatal and longer-term outcomes [3]. Fewer than half of pregnant women in the US eat the 200 mg/day of DHA recommended for optimal maternal and child health [2-4].

However, fish also may be contaminated with methylmercury, a ubiquitous toxicant present in all fish, especially long-lived, predatory fish [5]. Approximately 10% of women of childbearing age in the US have mercury levels higher than the recommended level of 5.8 μg Hg/L in blood or 1.2 μg Hg/g in hair [5,6]. Some experts recommend an even lower threshold of 3.5 μg Hg/L blood to provide maximal protection against harm [7]. Because of the demonstrated neurotoxicity of methylmercury and the particular susceptibility of the developing brain even at low exposure levels [8], the US Food and Drug Administration (FDA) and Environmental Protection Agency (EPA) have issued warnings recommending that pregnant women limit their fish consumption and choose fish species that tend to have lower levels of mercury [9]. Pregnant women, and other US adults, consumed less fish after these guidelines were disseminated [10,11].

The overall influence of fish – including its component nutrients and toxicants – on maternal and child health outcomes remains uncertain. While several observational studies have demonstrated improved mood among mothers who ate more fish in pregnancy [12], and better neurodevelopment among their children [13-19], these studies are subject to confounding. Furthermore, large randomized trials of omega-3 fatty acid supplements have not shown benefits for these outcomes [20]. The discrepancy between the benefits of fish intake implied by the observational studies and the null results from trials of supplements might be explained by the many potential nutrient benefits of fish in addition to n-3 PUFA, including vitamin D, iodine, and selenium [2]. In fact, consumption of lean fish during pregnancy may provide as much if not greater benefit than fatty fish for perinatal outcomes such as fetal growth [21-23]. Alternatively, the inclusion criteria for supplement trials may have been overly stringent, so that some individuals at risk for adverse outcomes were already taking supplements, and thereby excluded [24].

A randomized controlled trial optimizing fish consumption is the most unbiased way to determine its impact on improved maternal and child health outcomes. However, it is yet not clear how best to promote fish consumption during pregnancy without increasing mercury exposure. The feasibility of such a nuanced public health message is uncertain. Standard risk communication principles suggest that simple public health messages (“don’t smoke”) are most likely to achieve the targeted behavior, but not all questions can be addressed with such simple messages [1]. We therefore developed and conducted a pilot randomized trial of advice to promote consumption of low-mercury, high-DHA fish. Our goal was to determine if a nuanced public health message (i.e. avoid high-mercury fish, but eat more low-mercury fish) could be implemented successfully. We hypothesized that, compared with controls, women randomized to receive the intervention would increase their intake of fish, and DHA from fish, without an increase in mercury intake.

## Methods

### Study design and population

In April-October 2010 we recruited women using postings displayed at Boston-area obstetrics clinics, advertisements in a local newspaper, online classified advertisements, and local parenting listservs. The posting identified the project as a study of “diet during pregnancy,” but did not mention fish. We provided a phone number and e-mail address for interested women to contact us. The Harvard Pilgrim Health Care Human Subjects Committee approved all study protocols and materials, and all procedures were in accord with the Declaration of Helsinki.

A research assistant interviewed responders via telephone to describe the study in detail, screen for eligibility, and collect demographic information. To be eligible for participation, a woman had to be at least 18 years of age, have a singleton pregnancy at <=22 weeks gestation, and plan to remain in the Boston area through delivery. Because we were interested in targeting women with low fish and DHA intake, we included only women who reported consuming fish up to 2 times per month, and who had no contraindications to fish consumption such as allergy, or self-restrictions such as vegetarian diet. So that potential participants were not aware of the study’s particular focus on fish, the screening questionnaire also queried other components of diet including intake of fruits and vegetables, dairy, nuts, and meat.

**Figure 1:**
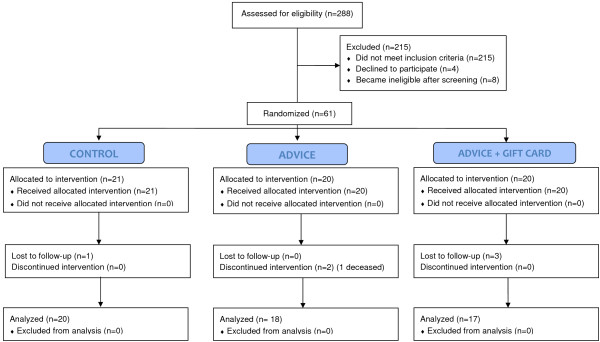
**Flow diagram of Food for Thought participants from recruitment to 12**-**week follow**-**up.**

Of 288 respondents (Figure 1), 215 did not meet inclusion criteria, most often because they reported consuming fish more than twice per month (n=155, 72%) or were already at least 22 weeks gestation (n=34, 16%). Only 4 women declined participation, and another 8 subsequently became ineligible (by failing to schedule the baseline study visit before 23 weeks gestation, having a miscarriage, or learning of a twin gestation). We conducted baseline visits at 12–22 weeks gestation with 61 women. One woman disenrolled after randomization, but before study recruitment was complete, and therefore we increased our targeted sample size from 60 to 61. Subsequently 4 women were lost to follow-up, and one died. Therefore, we obtained baseline and follow-up information on 55 women.

### Baseline measures and randomization

At the baseline study visit, which we conducted in our research offices in Boston or at the participants’ home or office, the research assistant obtained written informed consent and administered an interview to collect information on pregnancy history, health, and socio-demographic characteristics. Participants completed an Edinburgh Postpartum Depression Scale (EPDS) [25]. We queried the type, dose, and frequency of supplements taken. We collected blood by venipuncture.

We assessed fish intake using 36 questions that queried intake of 25 specified types of finfish and 11 types of shellfish/bivalves. We based our instrument on one used in the National Health and Nutrition Examination Survey (NHANES) [6,26,27], and we also included an additional 4 fish types likely to be consumed in our geographic area, based on our preliminary research [28]. We asked women to report the number of times they had eaten each of the 36 fish types during the previous 30 days. In addition, because the NHANES instrument did not obtain information on serving size, we also asked women to report the number of ounces per serving of each type for which they reported any consumption. We provided reference food models for 1 and 3-ounce serving sizes to support accurate reporting. We asked women who had reported consumption of canned tuna whether they had eaten white, chunk light, or both.

We also assessed general diet using PrimeScreen, a brief validated [29] food frequency questionnaire (FFQ) that includes 21 questions on diet during the past 30 days, including 4 questions on intake of “Canned tuna fish,” “Dark meat fish, for example mackerel, salmon, sardines, bluefish, or swordfish,” “Other fish, for example cod, haddock, or halibut,” and “Shrimp, lobster, scallops, or clams as a main dish.” Six response categories ranged from “never” to “nearly daily or daily.” These questions were similar to those used in a comprehensive semiquantitative FFQ that has been extensively validated in pregnant and non-pregnant adults, and used to examine associations of dietary fish and elongated n-3 PUFA intake with a large number of health outcomes (e.g. [4,14,30-34]).

We prepared sealed opaque envelopes in which we included study intervention materials for each of the 3 arms, and subsequently ordered them using a random number table. After all baseline measures were collected, the research assistant opened the next sequentially numbered envelope, and spent approximately 30 minutes reviewing the contents with each participant, answering all questions. At the completion of the visit, we provided all women with a $25 gift card and a fabric shopping bag with the study logo.

**Figure 2:**
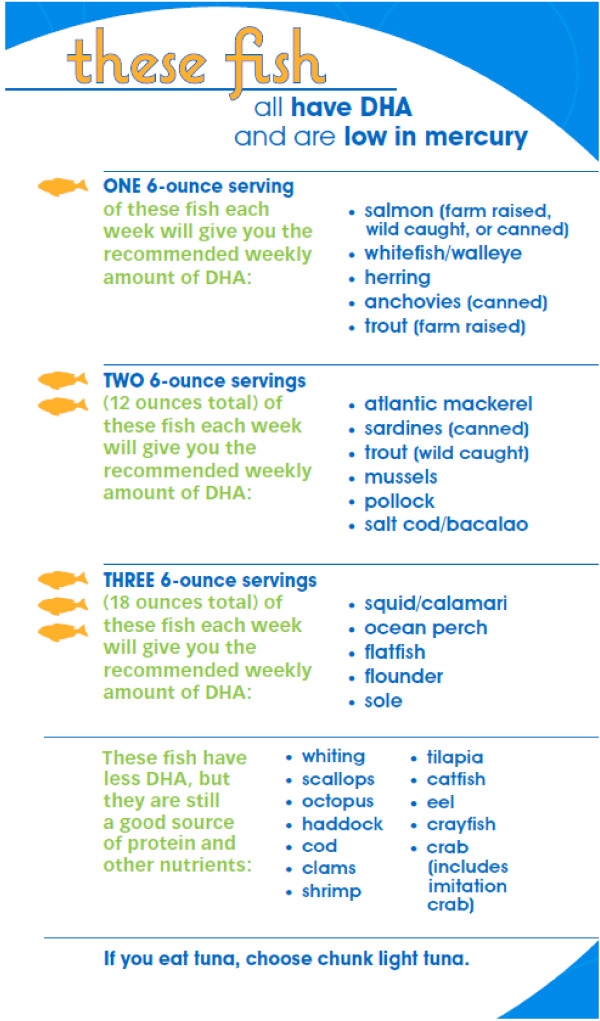
List of fish types provided to participants in the Advice and Advice+Gift Card arms.

For women randomized to the two intervention arms, we provided an 8-page booklet that summarized the health effects of DHA in pregnancy, encouraged fish intake, and included a list of 29 recommended low-mercury fish sorted according to DHA content (Figure 2). The booklet also included information on which fish to avoid to minimize exposure to mercury and other contaminants, based on advice from the US federal government [35] and Massachusetts Department of Environmental Protection (Figure 2). We also provided a shopping list notepad that included the list of recommended low-mercury fish ranked by their DHA content and 2 copies of a wallet-sized card summarizing the information in the brochure. We encouraged women to give the second copy to a partner or other family member who purchased food. We prepared these materials based on our preliminary qualitative research [28], targeted at a 5^th^ grade reading level, and pilot tested them prior to use with Boston-area pregnant women not enrolled in the trial.

Each week thereafter during the 12 week-long intervention, we emailed women in the intervention groups a “Weekly Thoughts” email, in which we encouraged consumption of 2 weekly fish servings, included a tip on the health benefits of fish or DHA, provided a recipe to prepare one of the fish types recommended in our brochure, and included a website address for more information.

Women randomized to the Advice + Gift Card arm also received a $40 Whole Foods gift card at the baseline visit, and we mailed them an additional gift card each of the 2 subsequent months, for a total of $120 ($10/week). We encouraged the women to use the gift cards to purchase fish.

We provided women randomized to the control arm with a 7-page “Pregnancy Food Guide” and 1-page list of “Food Don’ts,” both materials commonly given to women receiving prenatal care at local obstetric practices. These materials provided advice on a variety of nutritional topics for pregnant women, including advice based on the 2004 US governmental mercury advisory for pregnant women [35], to avoid the 4 fish types highest in mercury, and eat up to 12 ounces a week of a variety of fish and shellfish that are lower in mercury. After the baseline visit, we sent control women weekly emails with tips on general pregnancy health and recipes, not focused on fish.

### Follow-up measures

At the end of the 12-week intervention period we held an in-person follow-up visit (at a mean of 30 weeks gestation), at which participants completed self-administered questionnaires that included many of the same questions administered at baseline, including the same fish intake recall, use of DHA supplements and supplemented foods, and the EPDS. The follow-up questionnaire also included a series of questions about women’s opinions and attitudes regarding fish consumption, with 4-point Likert response scales ranging from “strongly disagree” to “strongly agree.” We asked women to report what they believed the focus of the study to have been, and coded the unstructured responses to indicate whether the woman mentioned fish or not. We again collected blood and also obtained a full-length sample of hair (“matchstick-thickness”) from the occipital scalp.

We provided an additional $25 gift card to all participants at the completion of the follow-up visit. About 2 weeks after the woman’s due date, we administered a brief questionnaire via telephone, mail, or email, to collect information on pregnancy duration and birth outcomes. We obtained post-delivery data from 48 of the 55 women who attended the follow-up visit.

### Biosample storage and assay

We refrigerated all blood samples immediately after collection, and processed and stored them at −80°C within 24 hours. We collected and stored whole blood in trace element-free tubes for mercury assay. We stored hair tied at the proximal end in paper envelopes at room temperature. We measured total mercury in whole blood and hair using the Direct Mercury Analyzer 80 (Milestone Inc., Monroe, CT). We analyzed the proximal 3 cm of hair, which reflects mercury deposited approximately 30–120 days prior to collection (the most recent 30 days’ growth remains below the scalp). Using standard samples, the Relative Standard Deviation (RSD) was 6.2% for blood (interlaboratory study program INSPQ, Laboratoire de Toxicologie, Quebec), and 2.4% for hair (Homogenized powdered hair, Institute of Geophysical and Geochemical Exploration, Langfang, China). We detected Hg concentrations as low as 0.3 mcg/g with a minimum sample weight of 0.2 g.

We collected whole blood in lithium heparin tubes, centrifuged it at 2,000 rpm at 4°C for 10 minutes, and stored plasma for fatty acid assay. Fatty acids were extracted and quantified in a single run using gas–liquid chromatography on a fused silica capillary cis/trans column (SP2560, Supelco Inc, Belefonte, PA) [35]. Peak retention times and area percent of total fatty acids were identified from 40 peaks by injecting standards (NuCheck Prep, Elysium, MN), using Agilent Technologies ChemStation A.08.03 software for analysis. With this method, the minimum reliable normalized percent area is 0.05%, and the within-run CV for DHA was 4.1%.

### Estimation of dietary fatty acid and mercury intake

We estimated daily intake of DHA from supplements based upon the dose and frequency of consumption. If the woman provided information on the brand of supplement but not the exact dose, we obtained DHA concentration from the manufacturer. For women (n=5) for whom we had information on DHA supplement frequency but not dose or brand, we assigned the median value of supplement DHA reported in the study population (200 mg/dose). For each of the 2 methods of dietary assessment, we calculated daily intake of DHA (mg/day) and mercury (mcg/day) from fish as detailed below. We also calculated intake of mercury per kg body weight per day. Within one week after the study visit, we contacted women with estimated mercury intake above the EPA reference dose of 0.1 mcg/kg body weight/day. We made specific recommendations to reduce their mercury intake based on their dietary report.

To assign a DHA concentration to each fish type included in the fish questionnaire, we used the USDA Nutrient database [36]. For mercury, we used data provided by the FDA [37], or from a publication by Groth [38] for fish types not included on the FDA website. We multiplied the number of servings by the reported serving size for each fish type, and summed intake across all fish consumed to estimate total daily DHA and mercury intake from fish. In our analysis, we defined low-mercury, high-DHA fish as those containing >400 mg DHA/100 mg of fish and <0.1 mcg Hg/g of fish, which included salmon, whitefish, herring, anchovies, trout, mackerel, sardines, mussles, pollock, and saltcod. We defined higher-mercury fish as those containing ≥0.1 mcg Hg/g fish, which included lobster, tuna, swordfish, shark, seabass, porgy, perch, and bass.

For the 4 PrimeScreen questions on fish, we applied weightings for the different fish types included in each of the 4 groups, based on the study participants’ eating habits from the 36-item questionnaire results. For example, “dark meat fish” was 20% anchovies, 1% mackerel, 64% salmon, 14% sardines, and 1% trout. We assumed a 113 g (4 ounce) serving size for each of the groups, based on the response categories used in the parent SFFQ from which we derived our instrument [39,40]. For each serving, we assigned concentrations of DHA (460 mg for canned tuna, 1406 mg for dark fish, 313 mg for other fish, and 232 mg for shellfish) and mercury (25.8 mcg for canned tuna, 2.2 mcg for dark fish, 4.8 mcg for other fish, and 2.4 mcg for shellfish.

### Blinding

All study staff and participants were blinded to group assignment before baseline measures were collected. To minimize bias introduced by non-blinding of the single research assistant, who both delivered the intervention and collected follow-up data, all follow-up self-reported data were collected by self-administered questionnaire rather than by interview. Laboratory staff, statistical analysts, and study investigators remained blinded to group assignment throughout data collection and analysis.

### Data analysis

We performed an intention to treat analysis including all women on whom we obtained data at the follow-up visit. We used 1-way ANOVA analyses to compare differences in change from baseline to followup within each of the 2 intervention groups vs. the control group for the dietary and blood measures of fish, fatty acid, and mercury intake. We also compared hair mercury at follow-up in the intervention groups vs. the control group using the same method. We used Fisher’s exact analysis to compare differences in dichotomous (clinical) outcomes in the 2 intervention groups vs. the control group. Our primary analyses were unadjusted; additional adjustment for baseline characteristics and gestational age at baseline or follow-up did not change results, and therefore we present unadjusted results only. We performed all analyses using SAS Version 9.3 (Cary, NC).

## Results

Maternal age (mean 30.2 [SD 5.6] years), pre-pregnancy BMI (25.6 [6.1] kg/m^2^), and other characteristics did not differ among groups at baseline (Table 1), although the proportion of women working full-time was somewhat higher in the Advice + Gift Card group (50%) than in the other two groups (35%). Estimated mean (range) intakes of fish [21 (0–125) g/day], DHA from fish [91 (0–554) mg/d], and mercury from fish [1.4 (0–8.5) mcg/day], were not different across the 3 arms (Table 1), although DHA intake from fish was somewhat higher in the Advice group (mean 132 mg/day) than in the Advice + Gift Card (79 mg/day) and control (63 mg/day) women. We saw no baseline differences in intake of DHA from supplements, plasma DHA concentration, or whole blood mercury levels across the 3 groups (Table 1).

**Table 1:** **Baseline characteristics of 55 women enrolled in the Food for Thought study**, **by study arm**

**Characteristics**	**Total (N=55)**	**Control (N=20)**	**Advice (N=18)**	**Advice + Gift Card (N=17)**
	**Median (IQR) or N (%)**			
Age (y)	30.9 (25.6, 33.8)	32.4 (27.7, 34.3)	32.6 (27.9, 35.9)	27.6 (24.5, 32.0)
Gestational age (wks)	16.1 (13.3, 20.7)	19.1 (14.7, 21.0)	15.2 (13.0, 18.6)	16.4 (13.9, 21.0)
Pre-pregnancy BMI (kg/m^2^)	23.4 (21.1, 28.3)	22.3 (21.1, 27.0)	25.8 (22.8, 34.5)	23.4 (20.7, 28.3)
Single	11 (20%)	4 (20%)	3 (17%)	4 (24%)
First pregnancy	16 (29%)	6 (30%)	6 (35%)	4 (22%)
Race/ethnicity				
White	27 (49%)	9 (45%)	9 (50%)	9 (53%)
Black	6 (11%)	2 (10%)	2 (11%)	2 (12%)
Asian	6 (11%)	3 (15%)	2 (11%)	1 (6%)
Hispanic/other	16 (29%)	6 (30%)	5 (28%)	5 (29%)
Working full time	22 (40%)	7 (35%)	6 (35%)	9 (50%)
Never smoker	40 (73%)	14 (70%)	14 (78%)	12 (71%)
**Supplements**				
DHA from supplements, all (mg/day)	0 (0, 200)	0 (0, 160)	0 (0, 200)	0 (0, 180)
DHA from supplements, takers* (mg/day)	200 (200, 275)	238 (200, 315)	200 (200, 200)	200 (180, 200)
**Diet**				
Fish intake (g/day)	11 (4, 23)	10 (4, 18)	15 (2, 37)	10 (7, 23)
DHA intake from fish (mg/day)	41 (11, 113)	40 (11, 63)	75 (6, 203)	40 (32, 111)
DHA from diet > 200 mg/day (%)	8 (15%)	1 (5%)	5 (28%)	2 (12%)
Total DHA diet + supplements (mg/day)	113 (36, 238)	62 (37, 203)	200 (24, 356)	116 (54, 235)
Mercury intake from fish (mcg/day)	0.6 (0.1, 2.0)	0.3 (0.1, 2.1)	0.7 (0.0, 1.8)	0.6 (0.2, 1.5)
Mercury intake (mcg/kg/day)	0.01 (0.00, 0.03)	0.01 (0.00, 0.03)	0.01 (0.00, 0.03)	0.01 (0.00, 0.02)
**Biomarkers**				
Plasma DHA (% of total fatty acids)	1.9 (1.5, 2.3)	1.8 (1.5, 2.2)	2.1 (1.8, 2.4)	1.8 (1.6, 2.2)
Whole blood mercury (mcg/L)	1.0 (0.7, 1.7)	1.1 (0.8, 1.5)	0.9 (0.6, 2.0)	0.9 (0.8, 1.9)
Whole blood mercury > 3.5 mcg/L† (%)	4 (7%)	1 (5%)	1 (6%)	2 (12%)

*Among those taking supplements (n=6 in each of the 3 arms). †Threshold per Mahaffey et al., 2009 [7].

IQR = interquartile range.

### Primary outcomes

At the follow-up visit there were substantial differences in dietary intake between intervention and control participants (Table 2). At follow-up, no control women consumed the recommended 200mg/d of DHA from fish, compared with 33% of women in the Advice group (p=0.005) and 53% of women in the Advice+GC group (p=0.0002). Over the 12 week intervention period, intake of fish increased in the Advice + Gift Card (+165 g/day) and Advice (+99 g/day) arms, but hardly changed in the control arm (+13 g/day). Intake of DHA from fish accordingly increased from baseline in the intervention arms (Advice + Gift Card: +148 mg/day, Advice: +63 g/day), but actually decreased a bit among control women (−11 mg/day). In contrast, DHA intake from supplements remained essentially stable in all 3 groups (Table 2).

**Table 2:** **Change from baseline to follow**-**up in intake and biomarker levels of fish**, **docosahexaenoic acid** (**DHA**), **and mercury**, **and hair mercury collected at follow**-**up**, **by study arm**, **among 55 pregnant women enrolled in the Food for Thought study**

**Change from baseline to follow-up**	**Control N=20**	**Advice N=18**	**Advice + Gift Card N=17**	**Advice vs. Control**	**Advice + Gift Card vs. Control**
	**Mean (SD) or %**			P value*	
**Supplements**					
DHA from supplements (mg/day)	10 (80)	10 (52)	34 (76)	0.99	0.31
**Diet**					
Fish intake (g/day)	2 (17)	14 (22)	24 (36)	0.15	0.01
DHA from fish (mg/day)	−12 (75)	57 (114)	149 (118)	0.04	<0.0001
DHA from fish + supplements (mg/day)	2 (11)	67 (130)	183 (144)	0.10	<0.0001
Mercury intake from fish (mcg/day)	0.4 (1.8)	−0.1 (2.1)	0.9 (1.8)	0.37	0.50
Mercury intake from fish (mcg/kg/day)	0.007 (0.03)	−0.003 (0.02)	0.012 (0.03)	0.27	0.55
**Biomarkers**					
Plasma DHA (% of total fatty acids)	0.01 (0.45)	−0.12 (0.66)	−0.07 (0.29)	0.03	0.52
Whole blood mercury (mcg/L)	0.35 (1.12)	−0.26 (0.36)	−0.40 (1.27)	0.67	0.22
**Follow**-**up**					
Hair mercury (mcg/g)	0.31 (0.31)	0.25 (0.23)	0.31 (0.30)	0.50	0.99

* P value from 1-way ANOVA.

**Figure 3:**
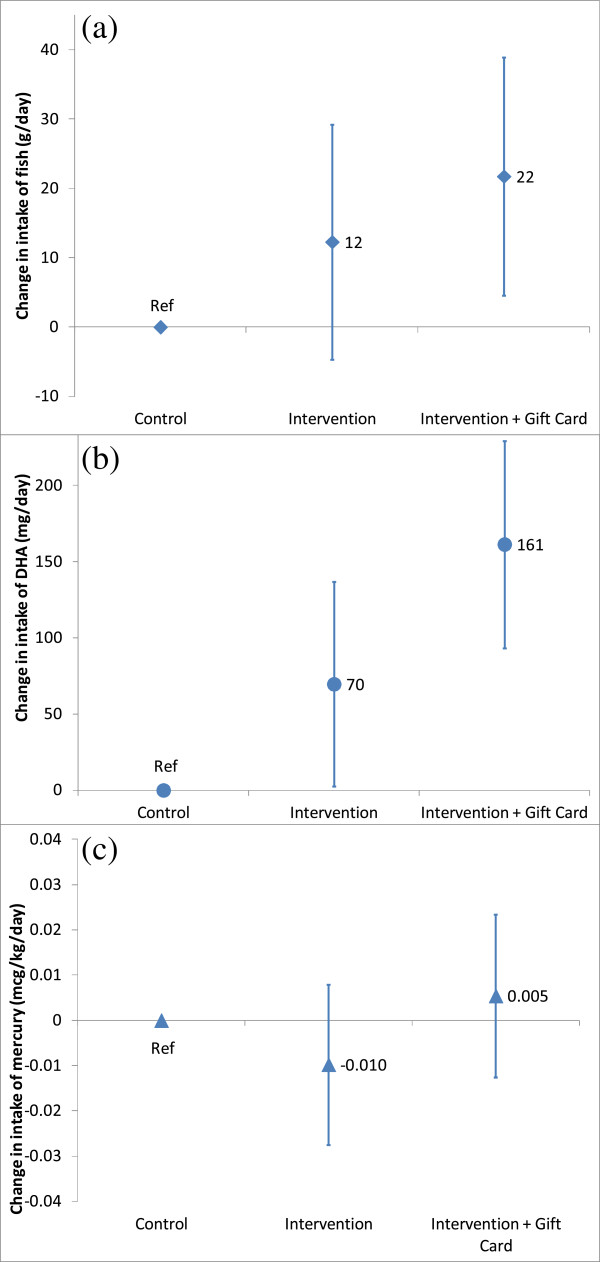
**Changes from baseline to follow-up among Advice and Advice +Gift Card vs. control participants in the Food for Thought study, for intake of fish (3a), docosahexaenoic acid (DHA) (3b), and mercury (3c).**

Compared with the control group, women in the Advice + Gift Card group had significant increases over the duration of the study in intake of fish (22 g/day, 95% CI: 5, 39) and DHA from fish (161 mg/day, 95% CI: 93, 229) (Figure 3). Women in the Advice-only arm had a somewhat smaller increase in intake of fish (12 g/day, 95% CI: -5, 29), but still a significantly increased intake of DHA (70 mg/day, 95% CI: 3, 137), compared with controls (Figure 3). Almost half of the increase in fish intake was attributable to an increase in the types we identified as low-mercury, high-DHA fish (Advice + Gift Card: 10 g/day, 95% CI: 5, 15; Advice: 5 g/day, 95% CI: 0.3, 10). We observed no differences in intake of higher mercury fish (Advice+ Gift Card: -0.5 g/day, 95% CI: -7, 6; Advice:-4 g/day, 95% CI: -10, 2). Despite the differences in estimated fish and DHA intake, we did not observe any difference vs. control in change in plasma concentrations of DHA (Table 2) or n-3 eicosapentaenoic acid (Advice: -0.18%, 95% CI: -0.37, 0.01; Advice+ Gift Card: -0.14, 95% CI: -0.33, 0.05).

Intake of mercury from fish did not change substantially in any group (Table 2), and there were no material differences in change from baseline to follow-up between intervention and control women (Figure 3). Also, there were no differences between groups in hair mercury concentrations at follow-up, or in change from baseline to follow-up in blood mercury (Table 2). Changes in intake of fish, DHA from fish, and mercury from fish were similar in magnitude and significance when we used measures derived from the 4-question FFQ, rather than from the 36-item detailed fish questionnaire (data not shown).

### Process measures

There were substantial differences between study arms in fish consumption attitudes assessed at the follow up visit. Compared with women randomized to the control group, women randomized to the two intervention groups were substantially more likely to strongly agree that they “enjoy eating fish” (40% vs. 10%, p=0.02), that “fish contains nutrients that are healthy for my baby” (74% vs. 35%, p=0.004), and that “some kinds of fish are better for me to eat than other kinds” (83% vs. 55%, p=0.03). Intervention women were more likely to strongly disagree that “I try NOT to eat fish because it might be harmful for me or my baby” (63% vs. 35%, p=0.05). There were no differences in attitudes about the expense of fish, the convenience of eating fish, or knowledge of how to prepare fish (data not shown).

Intervention women were somewhat less likely to report that their obstetrician had provided them with written information about fish intake during pregnancy (37% vs. 60%, p=0.10) or had discussed fish intake with them (31 vs. 55%, p=0.09) during the index pregnancy. There was no difference in consumption of foods with added DHA (49% intervention, 35% control, p=0.33).

When asked post-delivery what they believed to have been the purpose of the study, all but two of the intervention women reported that the focus had been fish, seafood, DHA, or omega-3 fatty acids; the remaining two women reported that the focus was on diet or eating habits but did not mention fish. Only one control woman mentioned fish; the remainder thought the study was about diet during pregnancy in general. Among the 17 women randomized to the Advice +Gift Card group, only 2 reported not having used the gift cards to purchase fish; one commented “I’m not a fish person” and the other that the fish was “too expensive.”

### Safety and pregnancy outcomes

Five women had estimated mercury intake above the EPA reference dose: two women (in the Advice arm) at the baseline visit only, one woman (in the control arm) at the follow-up visit only, and two women (one in the Advice arm and one in the Advice + Gift Card arm) at both baseline and follow-up. Among these women, mean (range) hair Hg was 0.53 (0.37-0.70) mcg/g. Similarly, only four women had whole blood mercury above 3.5 mcg/L at baseline and two at follow-up and there was no difference between treatment groups at either timepoint. One woman randomized to the Advice arm died during the intervention period; her partner reported that the death was related to her pre-pregnancy health, but did not provide an exact cause of death. One infant of a mother who was randomized to the Advice arm was stillborn.

Among the 48 women from whom we obtained post-delivery information, we observed no differences by intervention arm in rates of gestational diabetes mellitus, pre-eclampsia, gestational hypertension, induction of labor, cesarean delivery, or postpartum depressive symptoms (data not shown). However, there was a suggestion of a lower rate of preterm delivery (<37 completed weeks gestation) among intervention vs. control women (0/31 vs. 2/17 [12%] p=0.12).

## Discussion

In this pilot randomized controlled trial, we found that an educational intervention resulted in increased intake of low-mercury, high-DHA fish among pregnant women with low fish consumption at baseline. Women randomized to the intervention had significantly and substantially greater intake of DHA, but no greater mercury intake. There were no differences in use of DHA supplements, which we did not target in our intervention. Process measures suggested that women in the intervention groups absorbed the study’s messages and developed more positive attitudes about the health effects of fish. Women in the control group were effectively blinded to the study’s focus on fish.

This pilot study was not powered to examine clinical outcomes, and accordingly there were no differences in most studied pregnancy outcomes. However, we found some evidence for a decrease in preterm birth among intervention vs. control women, although this difference was not significant in this small sample. Prolongation of gestation with attendant reduction in rates of preterm birth is one of the most consistent findings of prior observational studies of fish intake as well as intervention studies of n-3 PUFA supplements during pregnancy [24]. This finding will need to be followed-up in a larger trial of fish consumption advice. While we observed two deaths (one stillbirth and one maternal death) in the Advice arm, we have no reason to believe these deaths were related to the study’s intervention messages. However, this finding will also merit additional evaluation in a larger trial.

Although we observed differences in estimated intake of DHA, we did not detect an increase in plasma DHA concentration among intervention vs. control arms. In fact, plasma DHA, which normally decreases throughout pregnancy, actually decreased more among women in the Advice arm compared with controls, even though reported intake of DHA was greater in the Advice arm. We anticipated that plasma concentrations would be more sensitive to short-term changes in diet than concentrations in erythrocytes, which survive for an average of 120 days. However, it is possible that plasma is not sufficiently sensitive to dietary change. Alternatively, because intervention women became aware of the study’s focus on fish and DHA intake, there may have been reporting bias.

There are several limitations to this study. All women resided in the greater Boston, MA area, and thus results may not be generalizable to women living elsewhere with different access to fish. However, the population was of diverse racial/ethnic and socio-economic makeup. By design we limited recruitment to low fish consumers, i.e. women who reported intake of <= 2 monthly fish servings. Nevertheless, estimates of daily intake of fish, DHA, and mercury in our study were similar to those among women of childbearing age in the nationally representative US National Health and Nutrition Examination Survey (NHANES) [2,6]. Also, mean blood mercury in our population (1.4 mcg/L) was similar to that in NHANES (geometric mean 0.8 mcg/L) [6], and plasma DHA concentration (1.9%) was similar to that in a large cohort of pregnant women also from Boston (1.9%) [14]. Thus, even though our population had similar estimated fish consumption to reference populations not selected on the basis of fish intake, we still observed effects of the intervention messages on intake. Follow-up after 12 weeks might not have been long enough to allow detectable changes in biomarkers. However, prior studies have detected changes in plasma DHA [41,42] and blood mercury [43] within 8 weeks following dietary interventions to increase n-3 PUFA or reduce mercury intake.

We designed the intervention brochure to be administered in the context of a research study, and the research assistant reviewed all of the intervention messages with the participants at an in-person visit. However, as the materials were targeted towards a low literacy level, it is possible that these materials might also be effective at increasing consumption of low-mercury, high-DHA fish in a clinical or public health setting. Intervention women randomized to receive grocery store gift cards reported using these gift cards to buy fish, and had higher fish and DHA intake compared with women who received the educational intervention alone. While such an incentive is not feasible for a public health campaign, future research studies might consider providing a similar incentive to maximize contrast between study groups.

## Conclusions

Despite the promise of marine n-3 PUFA in improving perinatal health outcomes and child development, randomized trials of supplements have generally not supported the positive findings of observational studies of fish consumption. Because the type and frequency of fish consumed tends to vary with factors such as maternal age, race/ethnicity, and place of residence [7], factors that might independently be associated with offspring development or other relevant outcomes, it is possible that sociodemographic confounding underlies the observed benefits seen in previous cohort studies. Alternatively, it is possible that consumption of fish, rather than supplements, is necessary to improve maternal and child health outcomes. Some have been reluctant to encourage pregnant women to consume fish regularly because of concern that they may exceed recommended intake of mercury. In this pilot study, however, we demonstrated that it is possible to promote consumption of fish that is low in mercury and high in DHA among pregnant women who are infrequent fish consumers. A larger study with longer-term follow up will be needed to demonstrate whether these differences in intake improve maternal and child health outcomes.

Perhaps the major potential confounder in previous research into the health effects of fish intake is the increased methylmercury intake that may occur from greater consumption of fish, especially larger or predatory fish. The adverse effects of methylmercury would tend to mitigate any benefits of fish consumption for outcomes important to population health including offspring neurodevelopment and adult cardiovascular disease [19]. Our study demonstrates that a nuanced public health message can be implemented to effect beneficial changes in diet. We have found that an intervention to increase fish consumption, when carefully directed toward fish with low mercury content, is feasible, setting the stage for future research into the health effects of fish consumption that will not be confounded by mercury exposure.

## Abbreviations

DHA: Docosahexaenoic Acid; EPA: US Environmental Protection Agency; FDA: US Food and Drug Administration; FFQ: Food frequency questionnaire; NHANES: National Health and Nutrition Examination Survey; PCB: Polychlorinated Biphenyls; PUFA: Polyunsaturated Fatty Acids; US: United States

## Competing interests

None of the authors has a conflict of interest to report.

## Authors’ contributions

Study conception and design (EO, LBG, SP, JH, MWG, SFO, DCB, ROW); acquisition of data (EO, LBG, AB, DNP); analysis and interpretation of data (EO, LBG, ROW); critical revision of manuscript (all); statistical analysis (EO, LBG); obtaining funding (EO, MWG, ROW); administrative, technical, or material support (LBG). Dr. Oken had full access to all of the data in the study and takes responsibility for the integrity of the data and the accuracy of the data analysis. All of the authors have read and approved the final version of the manuscript.

## Sources of support

This project was supported by the National Institutes of Health (R01ES016314, K24 HD069408), pilot project funding from the HSPH-NIEHS Center for Environmental Health (P30 ES000002) and the Harvard Clinical Nutrition Research Center (P30 DK040561), and by the Harvard Pilgrim Health Care Institute.
